# A critical evaluation of the EU-virtual consultation platform (CPMS) within the European Reference Network on Rare Endocrine Conditions

**DOI:** 10.1530/EC-22-0281

**Published:** 2022-09-16

**Authors:** E K White, I V Wagner, C van Beuzekom, V Iotova, S F Ahmed, O Hiort, A M Pereira

**Affiliations:** 1 Division of Endocrinology and Centre for Endocrine Tumors, Department of Medicine, Leiden University Medical Centre, Leiden, The Netherlands; 2 Department of Endocrinology & Metabolism, Amsterdam University Medical Centre, Amsterdam, The Netherlands; 3 Division of Paediatric Endocrinology and Diabetes, Department of Paediatrics and Adolescent Medicine, University of Lübeck, Lübeck, Germany; 4 Department of Paediatrics, UMHAT ‘Sveta Marina’ Varna, Medical University of Varna, Varna, Bulgaria; 5 Faculty of Medicine Division 2, Internal Medicine Endocrinology, Leiden University Medical Centre, Leiden, The Netherlands; 6 Office for Rare Conditions, University of Glasgow, Glasgow, UK

**Keywords:** Endo-ERN, CPMS, rare diseases, virtual consultations

## Abstract

In 2017, the European Commission installed 24 European Reference Networks (ERNs) for different categories of rare and complex conditions to facilitate cross-border health care via virtual case consultations in a secure Clinical Patient Management System (CPMS). The ERN for rare endocrine conditions (Endo-ERN) previously reviewed the CPMS, in which they detailed the difficulties physicians encountered with the system and proposed solutions to these that should enable the system to be used to a greater extent. This paper will further the endeavor of the first by performing a critical evaluation of the CPMS, assessing how these suggested improvements have been implemented, and if these have affected the usage of the system. The evaluation involves an assessment of CPMS usage statistics since its conception that takes into consideration the technical updates and the external factors that may have affected these, including data from a review survey following a training workshop for our new healthcare providers (HCPs) added in January 2022. It appears that the improvements made to the system since the first review, in particular the implementation of the Operational Helpdesk, have had a positive effect in increasing CPMS membership; however, the regular usage of the system continues to fluctuate. Several suggestions are made on how to further facilitate the use of CPMS by our members both individually and network-wide, by integrating CPMS activities with other network initiatives and further integrating these into national health care systems as well as looking for ways to measure patient satisfaction from the CPMS discussions outcomes.

## Introduction

**Figure 1:**
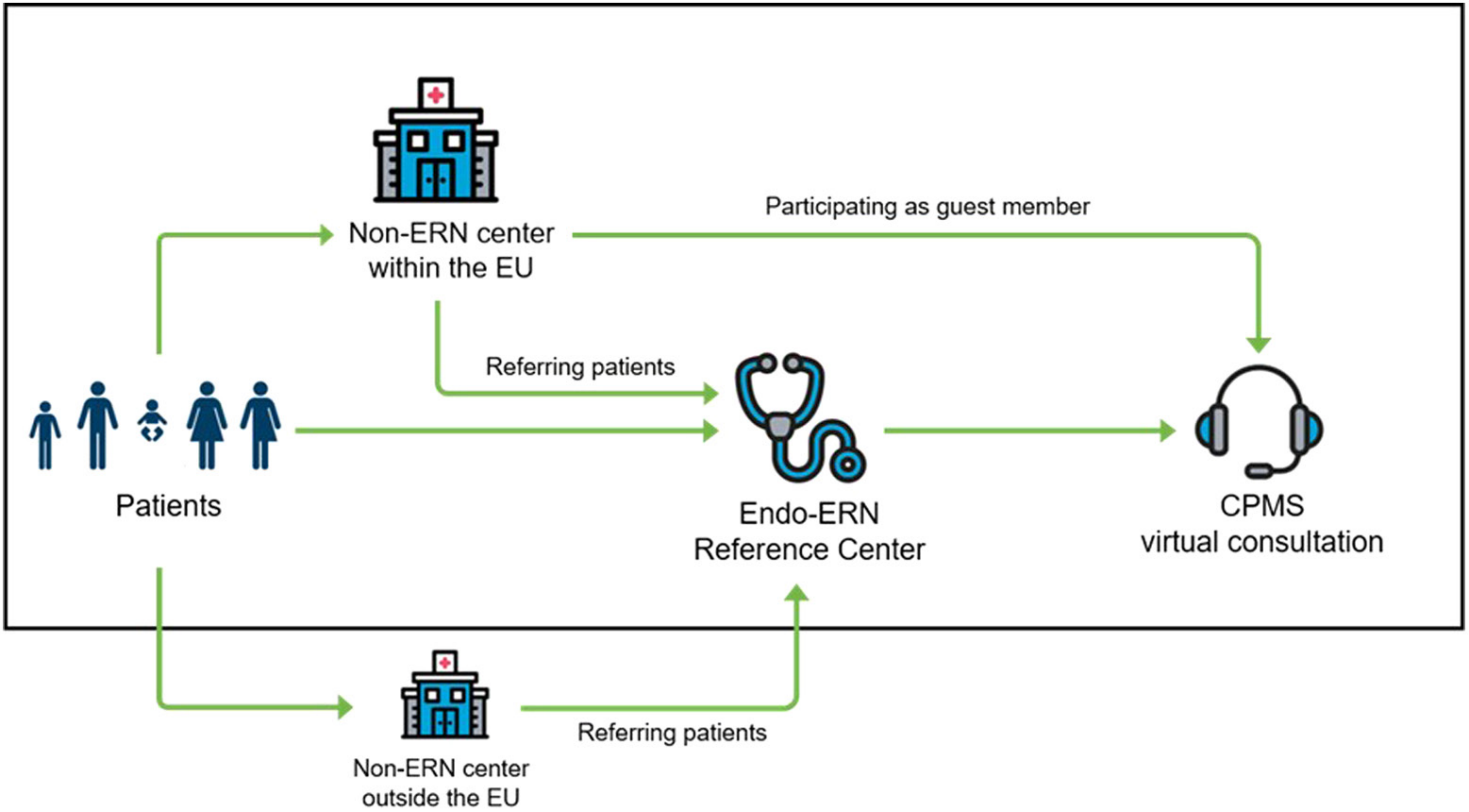
Flow chart of the different routes to have a patient case discussed in a CPMS virtual consultation. Modified, with permission, as depicted by Mönig et al. 2021 (3) from the Endo-ERN Clinical Patient Management System (https://endo-ern.eu/activities/clinical-activities-ehealth/cpms/). Copyright Endo-ERN CPMS Operational Helpdesk.

The Clinical Patient Management System (CPMS) is a secure web-based application which was specifically developed by the European Commission for the European Reference Networks (ERNs) for rare and complex conditions (1, 2). The CPMS is a secure web-based application for virtual consultations to provide expert specialized care for all rare endocrine disease patients within and across national borders (3, 4). Virtual consultations can take place since 2018. The CPMS platform is used to fulfill the ERN’s core task: to reduce health care inequalities within the European Union by providing access to expert specialized care to all patients with rare and complex diseases. The case discussions should lead to a standardized high-level care in all reference centers and allow expertise to travel instead of patients seeking individual care across the landscape (Fig. 1).

In total, 24 ERNs exist across 27 European countries. The European Reference Network on Rare Endocrine Conditions (Endo-ERN) is one of the largest ERNs with 111 reference centers as of January 2022 and 16 affiliated European Patient Advocacy Group representatives. Within Endo-ERN, eight different Main Thematic Groups (MTGs) are defined: MTG1 Adrenal; MTG2 Disorders of Calcium & Phosphate Homeostasis; MTG3 Genetic Disorders of Glucose & Insulin Homeostasis; MTG4 Genetic Endocrine Tumor Syndromes; MTG5 Growth & Genetic Obesity Syndromes; MTG6 Hypothalamic and Pituitary Conditions; MTG7 Sex Development & Maturation, and MTG8 Thyroid.

The CPMS is a virtual consultation platform, made specifically for ERNs, which complies with the General Data Protection Regulation (GDPR) of the European Union, and therefore includes appropriate measures for security and privacy for virtual consultations. Patients and their respective guardians where necessary need to provide informed consent prior to initiation of any discussion of anonymized data with relevant experts in that specific area. The development of the CPMS until 2020 within Endo-ERN was previously reported by Mönig *et al.* (3). To increase the use of CPMS for expert and complex patient care and to reduce barriers for experts to apply the system, an Endo-ERN-specific Operational Helpdesk (OH) was installed in 2020 to improve user-friendliness and help with technical difficulties and implementing CPMS to more healthcare providers (HCPs). After offering support through the OH, the number of registered users increased to 274 and the number of active users to 199 (3). However, Endo-ERN had the highest number of users per year in 2020 in comparison to all other ERNs, yet not the highest number of patient panels. The number of actively participating HCPs is increasing constantly but still too few experts use CPMS on a regular basis. The main issues reported by Mönig *et al.* for our users were technical difficulties, in particular the log-in process, lack of time, and insufficient awareness about CPMS. Despite this, members also expressed those virtual discussions with experts around Europe were very useful and helped to improve their patient care (3).

A year on from the first Endo-ERN CPMS review, the goal still remains the same – all involved experts should use and adopt CPMS into their regular practice, as well as promote the utility of CPMS and awareness of this resource as widely as possible amongst patients with rare endocrine conditions and their local physicians (3, 5). Therefore, in this paper, we critically evaluate the usefulness and effectiveness of the changes implemented to effect this change and critically analyze the system from the member’s perspective while considering additional factors that have arisen regarding the role of CPMS in Endo-ERN between April 2021 and 2022.

## Methods

The OH of Endo-ERN functions to continuously monitor the activities of our members and offer technical assistance with the CPMS when needed. Following the steps taken by the OH to increase user activity (3), we compared the activity trends between now and then. In October 2021, a review of user activity was conducted alongside the European Commission (EC) development team to optimize the system performance. Users with no activity in their accounts for a year or longer were deactivated, with reactivation possible only by contacting the OH. This allowed us to obtain a realistic indication of the CPMS activity within Endo-ERN. Additionally, it allowed the helpdesk to note and offer assistance to those experiencing difficulties. As the system continuously updates, manuals and instructive webinars created by the OH have been made publicly available for users. Additionally, the possibility to schedule a one-on-one dashboard tour for new and existing members has been advertised to members at the Endo-ERN General Assembly and via our Winter CPMS Newsletter. In January 2022, 34 new members were approved to join Endo-ERN. A CPMS training workshop was held as part of their introduction and welcome to the network. Following this, a review survey was distributed to all registrants, which included a follow-up query on a vote taken during the session itself on whether regular online walk-in sessions for CPMS would be beneficial to our members.

## Results

### Fluctuations of user and panel numbers between 2020 and 2022

**Figure 2:**
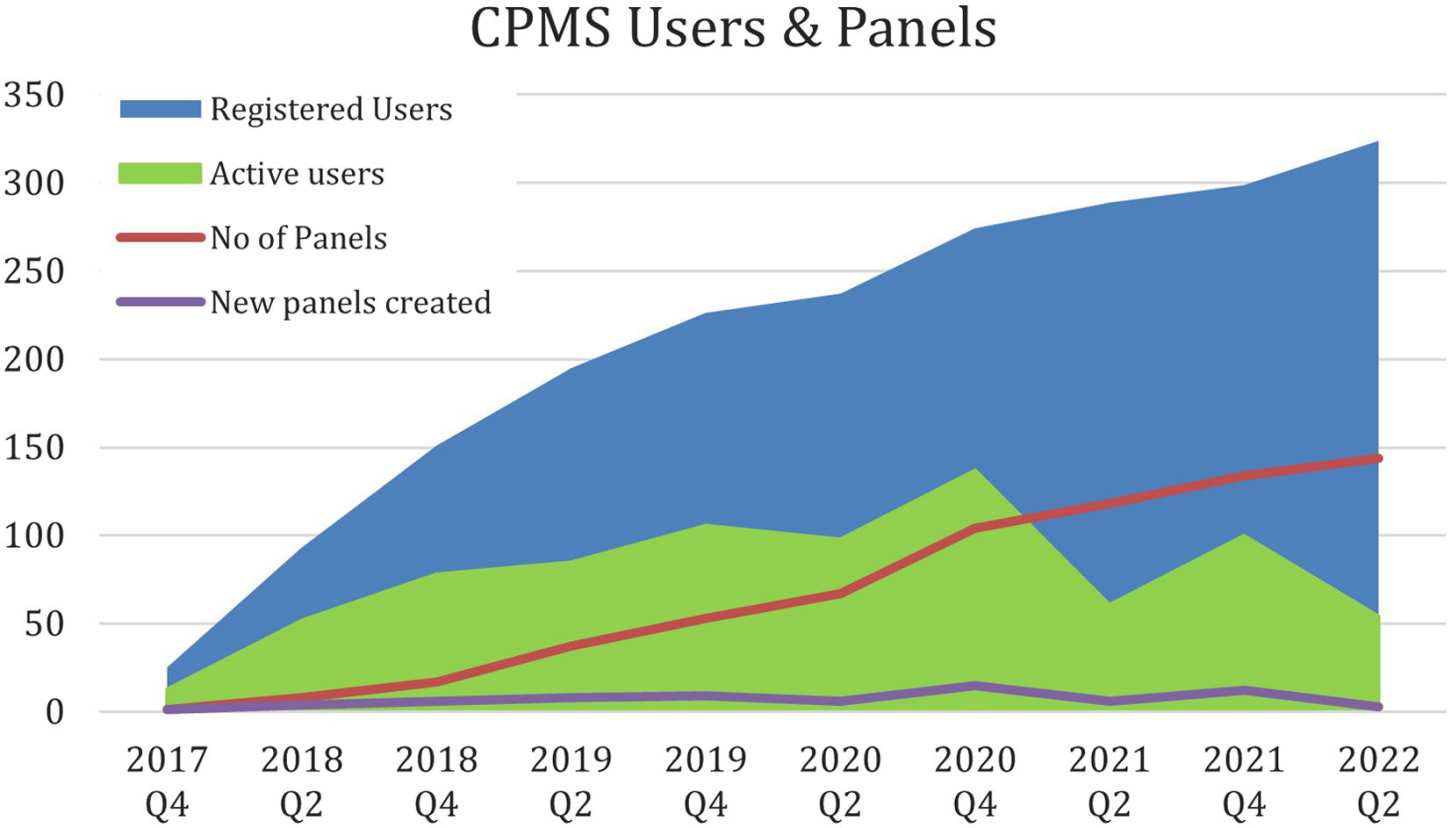
Record of total CPMS registered users, activity of users in each quarter, and total number of panels created since the system was activated.

**Figure 3:**
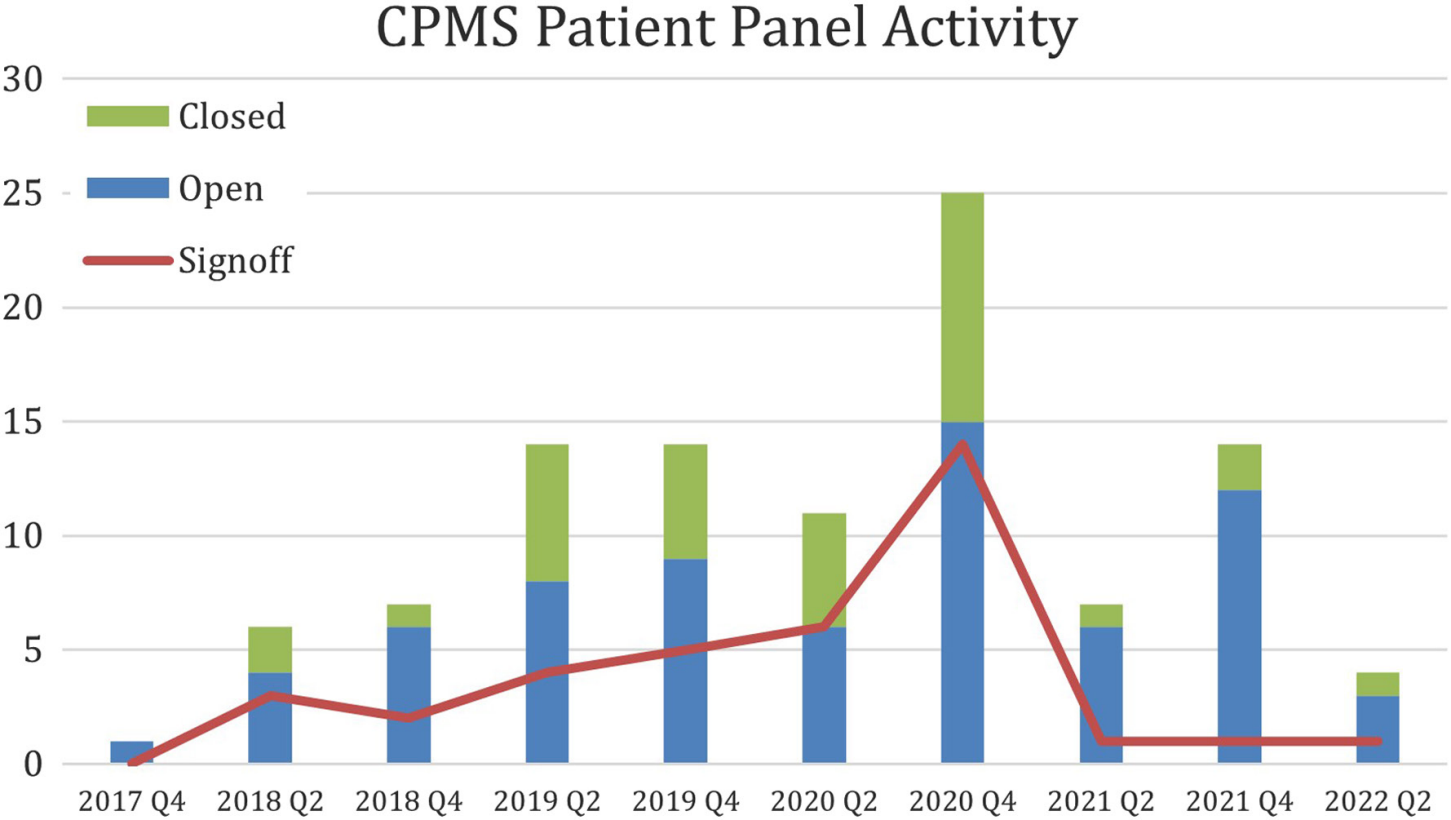
The trend of panel activity every 6 months since the system was activated, including the number of panels opened and closed out of the total 144 created. The number of panels signed off is noted here as the signing-off of a panel indicates the patient case outcome document is available.

In the first half of 2021, at the peak of the Covid pandemic, there was a shortage of staff and the OH was less active compared to the activity in 2020. Only 25 new members registered in 2021, the active number of users even decreased to 170 and only 30 expert panels were held. At present, the OH desk consists of two half-time working employees (health and medical psychologist and pediatric endocrinologist). Since the beginning of 2022, and hence in the first quarter, already 23 endocrinologists registered to the CPMS. The total number of registered users now enrolled in the CPMS is 326. At the moment, 270 users are regularly using the system with varying rates of activity each quarter (see Fig. 2). However, there remain 60 additional user accounts (excluded from these figures) which remain deactivated following the system review in October 2021. To date, there are 144 panels created on behalf of Endo-ERN in the CPMS in the first period from 2018 to 2022 (Figs 2 and 3). The panel activity has fluctuated since the start of the Endo-ERN.

The highest panel activity was registered in the second half of 2020 after the implementation of the different initiatives of the new OH (see also Fig. 3). The majority of the panels have also been closed and signed off. With new staff assigned to the OH, the panel activity increased again in the last 6 months of 2021, with the aim of re-gaining a similar number of panels in 2022 as in 2020. Activities of the OH targeting this goal included the increase and training of new CPMS members, acting as panel managers for newly created panels, and participation in ERN-CPMS workgroups to improve functionality in the new version of CPMS.

### Implemented technical updates and improvements of the CPMS to simplify CPMS usage

Technical changes made to the CPMS system between September 2021 and April 2022 followed as a result of collaborative efforts of ERN working groups to make the system more user-friendly. The time required to upload cases to the system was a user’s issue that acted as a specific barrier to use when the system was initially launched. Therefore, each CPMS working group, update, or system adjustment takes this into consideration to make the user’s experience more agreeable. The most recent updates included improvement of the chat interface, improvement of recurring meeting’s function, creation of Expert Groups in the CPMS, improvement of homepage and dashboard using color-coded area sections, and the development of customized patient datasets/records catering to the needs of an individual MTG. A significant change was also required to address some GDPR and data privacy red flags that arose in several member states regarding the CPMS. The data required for the first panels in CPMS were individually added and mandatory at first for all fields. This has since changed, where all addition of data is optional, beyond the basic required to identify the relevant expertise needed for a case. Additionally, it is less time-consuming to upload patient data which makes it more user-friendly. These adjustments have been agreeable to member states who had voiced concerns, additionally enabling them to approve of the use of CPMS in health care. As of January 2022, expansion of Endo-ERN includes such members, from Finland for example.

An updated CPMS User Manual was issued in January 2022 alongside scheduled dashboard alterations. This required an update of the existing manuals uploaded to the Endo-ERN website. Due to the numerous changes occurring, OH managers were invited from all ERNs to attend an informational webinar on the updates and to query any of the changes made. The increased number of roles in the CPMS is designed to lessen the burden on the panel lead to create, run, and add information to patient panels. The roles include the ability to select an HCP coordinator as a panel manager for a patient case alongside the panel lead.

### Initiation of training workshops and webinars to facilitate the usage of the CPMS

**Figure 4:**
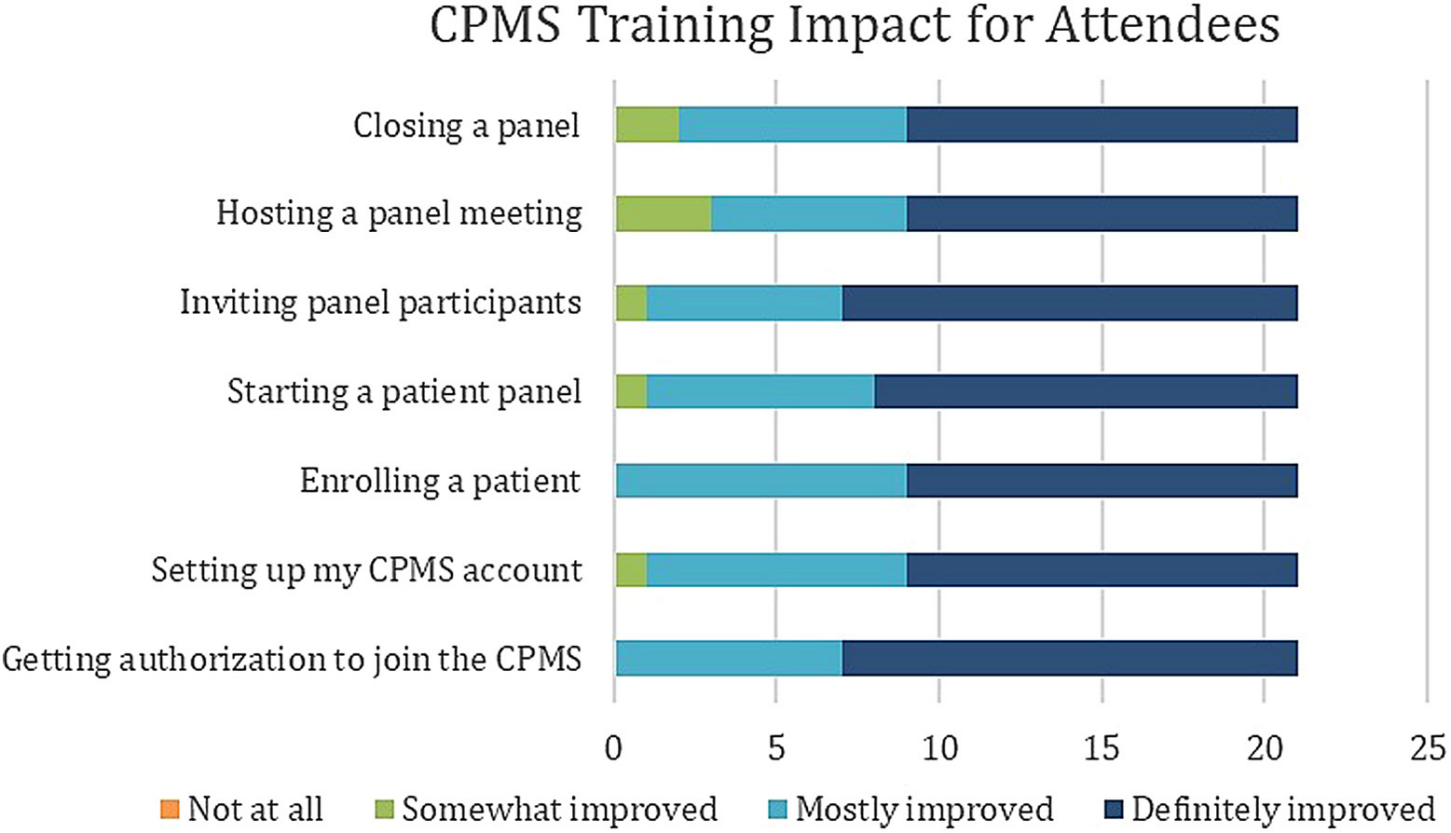
CPMS training impact on the improvement of capability in different core activities in a CPMS account, as reported by attendees.

The Endo-ERN OH ran a training workshop as part of the initiation of new members to the network. This workshop was open to Health Professionals in both new and existing member institutions, inclusive of our existing users within the CPMS. This was in part to ensure anyone with lower activity in the CPMS could improve their knowledge and comfort using the system. A virtual-based workshop was offered for new and registered members. A total of 78 applied to participate and 53 participants actively took part for the whole duration of the workshop. The workshop consisted of a guided tour through the CPMS going step by step through the core processes of setting up a CPMS account, enrolling a patient and running a patient panel, followed by how to close a panel appropriately. Afterward, 21 participants voluntarily evaluated the workshop in a review survey and the majority reported their knowledge on the core aspects of starting and using a CPMS account either as ‘mostly improved’ or ‘definitely improved’ (Fig. 4).

At the end of the workshop, a poll was taken in which participants were asked if a regularly scheduled designated virtual consultation for CPMS would be beneficial for clinicians every month. Of the respondents, 58% indicated that this would be useful for them while others noted that it could be useful to some clinicians, but for others, it would remain easier to schedule OH appointments only when an issue occurs or just before they want to start a panel in CPMS.

All ERNs have an affiliated patient registry. Specifically, for Endo-ERN, this is European registries for rare endocrine conditions (EuRRECA). In addition, the number of new patients seen every month through the accredited MTGs of an HCP can be registered electronically through a specific platform: e-REC. An update to the CPMS system has included the addition of a direct link between CPMS and the EuRRECa core registry database upon panel completion of patient data. Consent for data to be shared to the core registry must be obtained from the patient alongside that of the CPMS (6). This is also necessary to obtain for patient cases in CPMS if formal or informal educational activities are to be created using information from a discussed patient panel. Webinars on case topics and areas of interest, facilitated within the appropriate MTGs have been run for two patient cases so far in 2021 directly inspired by CPMS discussions. One additional webinar is scheduled directly for 2022 from CPMS currently. Further initiative to increase these case-specific webinars that can assist in improving the knowledge of rare endocrine diseases is the creation of standard operating procedures (SOPs) by the OH to schedule such webinars more easily. This is highlighted in one of the initiatives for the new EU4Health Endo-ERN grant period.

### Continuous monitoring program has been developed and will be applicable from 2023 for monitoring, evaluation, and improvement

The CPMS also plays a role in the mandatory continuous monitoring program and further development of Assessment Monitoring Evaluation and Quality Improvement System (AMEQUIS) protocols (7). The continuous monitoring program will begin to integrate these protocols in 2023. The AMEQUIS is being developed with the assistance of all ERNs by an external public knowledge association called Nivel (7). The continuous monitoring program is an initiative of the EC to appropriately evaluate ERN performance using key performance indicators (KPIs) and, furthermore, provide a roadmap through which an HCP’s ERN activities can be quantified. Two KPIs directly pertain to the CPMS. During the data collection period, HCPs report on CPMS panels they have collectively initiated and those they have partaken in separately. This initiated panel data collection will now also inform the CPMS pilot scheme recipients. The use of the CPMS by HCPs will be taken into consideration during monitoring and quality improvement steps.

## Discussion

Since the start of Endo-ERN, the number of clinicians who have registered to use CPMS is constantly increasing, but the number of active users and panels is not growing at the same rate. Therefore, to increase active participation, there is a need to understand and resolve the obstacles that are faced by health care providers. One of the main goals of the CPMS is to reduce inequalities in care for patients with rare endocrine diseases throughout Europe, can only be achieved if CPMS use is integrated into the national health care systems. When surveyed, all users rate the system as very useful and especially helpful to improve patient care, which questions why the number of panels and usage in general is not higher for the system, and which factors have contributed to the decrease in activity again after 2020.

The establishment of the OH did contribute, at least in part, to the observed increase in CPMS membership and use. An effective user support seems to require both roles in the OH to be filled as technical assistance can be offered more readily while running a panel or if issues are encountered determining which MTG experts should be involved. The OH offers both personal and group coaching sessions regarding the CPMS system to assist in overcoming all hurdles or technical barriers to using the CPMS in the care of patients with rare endocrine conditions.

In our system usage statistics (Fig. 2), we can see that the number of active users and panels has declined in 2021. Apart from the time factor which might be a strong hurdle to use the CPMS, the COVID-19 pandemic might have influenced the CPMS usage. On the one hand, clinicians were facing more work in hospitals with outpatients and COVID-19 patients and understandably did not have sufficient time to spend on individual patient cases with rare diseases beyond their usual workload. On the other hand, as people became more acquainted with telemedicine and virtual consultations, it is plausible to assume to see a reduction in anxiety in using the CPMS system. We speculate that this reduction in activity was partly due to a shortage of OH staff; hence, less support was available during this period. Users that are facing technical difficulties, or who did not reactivate their accounts yet since the system review last October, give up much more easily and prefer to quickly ask colleagues for advice on the phone or via e-mail without using the CPMS. As the OH for Endo-ERN now consists again of two half-time working employees, we anticipate to increase the user activity again in 2022, which is already reflected by the fact that 23 new users have registered to the CPMS in the first quarter of 2022.

Technical adaptations to the system made by the EC between September 2021 and April 2022 were implemented after working groups were conducted in a collaborative effort with ERNs to ask how to make the system more user-friendly. The Endo-ERN-specific customized patient consultation forms, including laboratory reference values, are now in the final test phases of their development for MTG 6 and MTG 7. The other MTGs will follow after the implementation for MTG 6 and 7 have been completed. The aim is to ease the process of entering patient’s data into the system as the amount of time required for the preparation of a case is an important consideration to use CPMS for any clinician. To ensure CPMS fulfills the criteria of being a secure web-based consultation platform across all member states, this data input will remain optional. Eventually, these improvements should result in an intuitive utilization of CPMS by the experts, and CPMS will become a standard item in their agendas. New functions can also lead to difficulties for the existing users and it takes time for them to adapt to changes, but this is an intrinsic feature of all electronic patient records. We tried to facilitate the usage of the updated CPMS system with a new CPMS user manual that was available from January 2022.

The improvements of CPMS are not only important for Endo-ERN but for all 24 European Rare Disease Networks. Therefore, the different CPMS helpdesks will continue to consult with each other and the technical support team DG SANTE on a regular basis. Once a month, all OHs of the 24 ERNs have a web-based meeting to discuss technical issues, improvements, and future plans. As previously reported by us, the registration process unfortunately is still perceived by many to be complicated and some technical problems remain at that step. There is an undercurrent of distress that remains after this process, which might negatively impact the experience of initiating any panel in CPMS, even if the procedure is straightforward and easier to implement than the registration process (3). As of March 2022, Endo-ERN has also elected to take part in a pilot incentive scheme created by the EC to encourage panel creation in the CPMS. The amount of €200 per completed panel is fixed and set by the EC; however, each ERN may decide to who this amount will be paid. Endo-ERN decided to pay the panel lead (instead of, for example, dividing the amount over all participating HCPs for the specific panel), in order to minimize the administrative burden. The scheme will hopefully assist with minimizing the burden on clinicians who work on these panels. After the panel lead completes a panel, they will be forwarded a survey to evaluate the benefit for HCPs. We will therefore be able to decide if this is an important offer to improve the panel numbers that were initiated, held, and completed.

Based on review survey and instant feedback, the participants found the Endo-ERN Helpdesk training workshop very useful. If members start using the CPMS regularly, technical issues should lessen. Some of the participants evaluated the CPMS as still being too complicated and found that the guided tour was too fast in their opinion. Therefore, we decided to offer workshops on a regular basis and in a practical step, we have enrolled a panel manager for each panel from the OH who will help with questions and technical problems during the setup and duration of the panel. Part of the problem is the lack of knowledge about the CPMS existence and the possibility of using it around Europe. For non-ERN experts, primarily treating physicians and of course the patients themselves, this could be an invaluable resource to access via their local ERN reference center. We anticipate that information will be easier to disseminate at meetings and conferences once the COVID-19 pandemic restrictions are lifted. The pandemic has also shown that video meetings do allow fruitful scientific and patient discussions, and telemedicine has been more and more implemented in patient care and in daily work routine.

Patient panels in the system and in rare cases are now acting as a support to peer learning for experts and assist in increasing awareness of the CPMS across Europe through affiliated organizations. Further initiative to increase case-specific webinars that can assist in furthering the knowledge of rare endocrine diseases is the creation of SOPs by the OH to schedule such webinars more easily. This is highlighted in one of the initiatives for the new Endo-ERN bridging grant, as part of the EU4Health Program.

A major challenge of the CPMS, which has not been addressed yet, is the lack of a measure of the patient benefit of the CPMS. There is no direct connection to the patient evaluations and interviews have not taken place. The clinical value of the system for the patients is harder to gauge without such measures and for now we must rely on the feedback of the expert clinicians who treat and discuss these patients, and our patient advocacy groups (1). This serves to further highlight the need for the national health care systems to integrate CPMS, also because virtual consultations will remain considerably cheaper than physical cross-border referrals for patients. Hence, preferential use of CPMS will effectively save national health care resources in the long run. Consequently, national healthcare systems in all member states will need to adopt and enforce a ‘CPMS first’ policy for any rare disease case, for which cross-border healthcare is considered.

In conclusion, our overview demonstrates the advantages and improvements that have been made in the last year, as well as highlights and discusses challenges and existing problems that have to be overcome in the near future. In general, participants evaluate the CPMS as being very useful and secure in video-based patient discussions, and the expert opinions are extremely helpful for good patient treatment in rare endocrine diseases. The main obstacles for frequent use are still technical difficulties and the time-consuming factor experienced by the HCPs. Joint efforts to support experts, to technically improve the system and to make it generally known among European patients and experts, can lead to a more regular use of CPMS and move closer to supplying expert specialized care for all patients with rare conditions in Europe in the (near) future. Though, during the future development of CPMS, it needs to be critically evaluated whether CPMS will prove its promise for the patients to bridge the longstanding disparities in healthcare within the EU.

## Declaration of interest

The authors declare that there is no conflict of interest that could be perceived as prejudicing the impartiality of this review.

## Funding

This publication has been supported by Endo-ERN, which is co-funded by the European Union’s 3rd Health Programme (CHAFEA Framework Partnership Agreement No. 739527). The current work is supported by a grant under the Connecting Europe Facility http://dx.doi.org/10.13039/100013294 (CEF) sector, agreement No INEA/CEF/ICT/A2020/2396778. This publication is supported by the European Reference Network on Rare Endocrine Conditions (Endo-ERN). Endo-ERN is funded by the European Union within the framework of the EU4H Programme, grant agreement No. 101084921.

## Author contribution statement

E K W and I V W have drafted the paper. S F A, V I and C v B have contributed aspects to the content, according to their expertise and function within Endo-ERN. O H and A M P have supervised the writing.
